# Psychosocial Impact of a True-Positive, False-Positive, or Inconclusive Newborn Bloodspot Screening Result: A Questionnaire Study among Parents

**DOI:** 10.3390/ijns10010018

**Published:** 2024-03-05

**Authors:** Lieke M. van den Heuvel, Sylvia M. van der Pal, Rendelien K. Verschoof-Puite, Jasmijn E. Klapwijk, Ellen Elsinghorst, Eugènie Dekkers, Catharina P. B. van der Ploeg, Lidewij Henneman

**Affiliations:** 1 Department of Human Genetics, Amsterdam UMC, Vrije Universiteit Amsterdam, 1007 MB Amsterdam, The Netherlands; 2 Amsterdam Reproduction and Development Research Institute, Amsterdam UMC, 1100 DD Amsterdam, The Netherlands; 3 Department of Child Health, Netherlands Organization for Applied Scientific Research TNO, 2333 BE Leiden, The Netherlands; 4 Department for Vaccine Supply and Prevention Programmes, RIVM Dutch National Institute for Public Health and the Environment, 3720 BA Bilthoven, The Netherlands; rendelien.verschoof@rivm.nl; 5 Centre for Population Screening, RIVM Dutch National Institute for Public Health and Environment, 3720 BA Bilthoven, The Netherlands

**Keywords:** newborn screening, abnormal result, inconclusive result, psychosocial impact, child vulnerability, parental perceptions, questionnaire study

## Abstract

Expansion of newborn bloodspot screening (NBS) can increase health gain for more children but also increases the number of false-positive and uncertain results. The impact of abnormal and inconclusive NBS results on parental well-being and healthcare utilization was investigated. A questionnaire was sent to Dutch parents receiving an abnormal or inconclusive NBS result five weeks (T1) and four months (T2) post-NBS and compared to parents with a normal result (controls). In total, 35 true-positive (TP), 20 false-positive (FP), and 57 inconclusive (IC) participants and 268 controls filled out T1; 19 TP, 14 FP, 27 IC, and 116 controls filled out T2. Participants showed positive attitudes towards NBS. FP participants more often considered NBS less reliable. TP and FP participants experienced more negative emotions regarding the test result compared to controls at both T1 and T2, and IC only at T1. Parent-reported child vulnerability and perceptions of the newborn’s health status and of parenthood showed no differences. TP and FP participants reported more healthcare utilization at T1, and mainly TP at T2. TP and IC participants showed more emergency department visits at T1. The findings can be used to improve NBS programs and optimize support for families with various NBS results.

## 1. Introduction

The aim of newborn bloodspot screening (NBS) is the early detection of rare yet serious congenital disorders in infants, allowing for timely treatment to avoid irreversible health damage. Technological advances continue to improve the clinical understanding of disorders and the availability of treatments, leading to an ongoing expansion of the number of disorders included in NBS programs [1,2]. While this expansion increases health benefits for a growing number of newborns, it raises several psychosocial and ethical issues [3,4,5,6]. 

One concern is an increase in the number of newborns referred for further evaluation due to abnormal NBS results, which later turn out to be false-positives. This increase in false-positive results, along with inconclusive results that necessitate a second heel prick or results that remain uncertain even after follow-up diagnostic testing, can potentially negatively impact the public’s perception and acceptance of NBS [5,7]. Additionally, these results may have a negative impact on parents’ psychosocial well-being and the development of involved children [5,8,9]. Some parents feeling disillusioned with the screening process may choose to opt out of future screening altogether. Given the current global trend of expanding NBS programs, a trend also observed in the Netherlands, it is crucial to address these issues promptly and comprehensively. 

Previous research on the psychosocial effects of false-positive NBS results has yielded mixed results regarding the intensity and duration of their impact on parents [8]. Some studies suggest that an abnormal NBS result, later determined to be a false-positive, can lead to adverse outcomes, including increased parental anxiety [5]. Earlier investigations have indicated persistent distress among parents of newborns [10,11], which may stem from inadequate information, understanding, or the timing of confirmative testing [8]. Moreover, research has hinted that false-positive results may impact perceptions of the child’s health [12], the parental–child relationship [13], and a temporary increase in the utilization of healthcare services [14,15,16]. Other studies, however, have found limited psychological impact [17,18,19,20] and reported no discernible increase in healthcare utilization among parents [21,22]. 

In the Netherlands, the acceptance of newborn screening by parents is notably high [23], with participation in the NBS program, which includes 27 disorders, exceeding 99% [24]. However, there has been limited research on the impact of abnormal results, including false-positive results, with most studies focusing on specific disorders such as cystic fibrosis [20] and severe combined immunodeficiency [25]. Overall, there is a scarcity of research that compares the impact of abnormal and normal NBS results within expanded NBS programs [5,19]. Gaining insight into parental experiences, including psychological impacts and their healthcare utilization when confronted with an abnormal NBS result, can provide valuable information to shape current NBS practices and guide decision-making regarding further expansion of NBS. This study, therefore, aimed to assess the impact of an (initially) abnormal or inconclusive NBS result on parents’ self-reported psychological well-being and healthcare utilization by comparing the impact among parents receiving a true-positive, false-positive, or inconclusive result with parents receiving a negative (normal) result within the Dutch screening program.

## 2. Materials and Methods

### 2.1. Design

A cross-sectional questionnaire design in a case-control cohort was used to assess the short-term (five weeks after NBS) and long-term (four months after NBS) psychosocial impact of an abnormal or inconclusive NBS result in the Netherlands. The study was conducted between October 2021 and April 2022. 

### 2.2. Setting

In the Netherlands, the Dutch National Institute for Public Health and the Environment (RIVM) is responsible for organizing and overseeing the national NBS program. To ensure parents are well-informed about the screening process, they receive oral information from the obstetric care provider, and they receive a leaflet during pregnancy and at the time of birth registration. It is important to note that participation in the NBS program is voluntary in the Netherlands. The program presently (January 2024) screens for 27 disorders, including various metabolic disorders, congenital hypothyreoidy, adrenogenital syndrome, cystic fibrosis, spinal muscular atrophy, three types of hereditary anemia, and SCID (Severe Combined Immunodeficiency) [24]. Since March 2020, all parents who receive a negative (normal) result also receive a confirmative letter within five weeks.

In the case of an abnormal NBS result, the RIVM’s medical advisor discusses the result with a specialized pediatrician and communicates the abnormal result and the referral details to the local general practitioner. Subsequently, the general practitioner discloses the result to the parents and refers the child to a hospital for further diagnostic tests. 

In the case of an inconclusive result, a second heel prick is recommended as the next step in the screening process.

### 2.3. Participants and Procedure

Three groups of parents were approached for this study: (i) those who received abnormal results from the NBS test, (ii) parents whose children received an inconclusive (IC) result, altogether referred to as “cases”, and (iii) parents whose children received a negative (normal) NBS test result, serving as “controls”. The group of parents who received abnormal results was categorized as either a true-positive (TP) or false-positive (FP) based on the questionnaire completed by the parents.

A true-positive result was defined as an abnormal NBS result that was subsequently confirmed as accurate after hospital referral, indicating that the child indeed had the specified condition. A false-positive result was defined as an abnormal NBS result that, following referral, was not confirmed by the presence of the condition in the child. An inconclusive result meant the first heel prick was inconclusive, necessitating a second heel prick, which subsequently produced a normal test result. Exclusion criteria were defined as parents of children identified as carriers of sickle cell disease (an incidental finding in NBS), parents living abroad, and parents of deceased children. 

Participants who received abnormal NBS results that led to a hospital referral (TP or FP) or inconclusive NBS results were sourced from Praeventis, the Dutch registration system maintained by the RIVM [24]. This database does not record the final diagnosis after an abnormal NBS result (which is recorded in another database that was not accessible for this study). Therefore, we relied on parents to report whether the result was TP or FP. For each case, three consecutive controls with negative (normal) NBS test results were invited to participate. 

The RIVM facilitated the study by sending out mailings to parents, including a letter of invitation from the researchers explaining the purpose of the study, a paper questionnaire, and a link to an online version of the questionnaire. An English version was provided upon request. Due to privacy considerations, the mailings were sent by the RIVM rather than the researchers, and it was not possible for the researchers to send reminders. 

Following completion of the first questionnaire (T1), parents were invited to participate in a second, shorter questionnaire approximately three months later. For the second questionnaire (T2), a maximum of two reminders were sent to parents who had enrolled in the study. To encourage participation, ten online gift cards worth €10 were randomly allocated to study participants. Before parents completed the (online) questionnaire, they were asked to tick a box (written consent) to confirm that they had read the information letter and that they gave their informed consent to participate in the study.

### 2.4. Measures

The questionnaire used in this study was developed based on the literature [19,20] and incorporated with input from professionals with relevant expertise. It included both self-constructed items and validated questionnaire items, covering three main topics: (1) perceptions of NBS, (2) the psychosocial impact of NBS results, and (3) self-reported healthcare utilization (see Supplementary S1: Questionnaire). 

The measures obtained per topic are described below. Cronbach’s alpha values were reported for measures to assess the internal consistency and item scores were summed up to sum scores if this value was at least moderate (α ≥ 0.6).

(1) Perceptions of NBS (T1 and T2): Participants in both the cases and controls were asked to respond to three self-constructed items related to their perceptions of NBS. Parents were asked to indicate on a 5-point scale whether they felt adequately informed about the heel prick test prior to having it (1 = certainly not—5 = certainly), their overall perception of the heel prick experience (1 = bad—5 = good), their perception of the reliability of the heel prick test (1 = not reliable—5 = reliable), whether they regretted participating in the heel prick (1 = a lot of regret—5 = no regret), and whether they intended to participate in NBS in a future pregnancy (1 = certainly not—5 = certainly). Parents who received an abnormal NBS test result (TP or FP) were additionally asked whether they felt satisfied with how they received the initial NBS result (1 = not at all satisfied—5 = very satisfied) and their satisfaction with the follow-up testing at the hospital. Parents with an IC result were asked whether they felt satisfied with the information provided regarding the second heel prick (yes/no; why not (open text field)).

(2) The psychosocial impact of the NBS result (T1 and T2) was assessed using various measures. Participants were asked how they felt about the (first) NBS result (including the request for a second heel prick, if applicable) and about the definitive result after follow-up testing, if applicable. Participants’ emotions related to the NBS result at T1 and T2 were measured using seven self-constructed items with a 5-point semantic differential scale (i.e., not reassured—reassured, startled—not startled, worried—not worried, anxious—not anxious, unhappy—happy, angry—not angry, and not relieved—relieved). Cronbach’s alpha for these items was high (0.96 (T1) and 0.93 (T2). Sum scores ranged from 0–35, with higher scores indicating more positive emotions. 

Symptoms of anxiety and depression were assessed using four items from the Hospital Anxiety and Depression Scale (HADS), three of which pertain to the anxiety construct and one to depression [26], based on Vernooij et al. [20]. The three items of the anxiety construct showed a moderate Cronbach’s alpha (alpha = 0.69), with a sum score ranging from 0 (low anxiety) to 9 (high anxiety). 

Parents’ perceived vulnerability of their child was assessed using items from the Child Vulnerability Scale (CVS) [27], which asked participants to agree or disagree with four statements on a 4-point scale (1 = totally incorrect—4 = totally correct) [28]. Cronbach’s alpha was shown to be moderate (0.63), and a sum score was reported ranging from 4 (low child vulnerability) to 16 (high child vulnerability). 

Perception of the newborn’s health status was assessed using nine items from the TAPQoL [29], covering aspects like physical complaints, sleep problems, crying at night, appetite, and the child’s emotional state. All items had three answer options (never, sometimes, and often) [30]. Cronbach’s alpha was shown to be moderate (0.63); sum scores were reported ranging from 9 (good health status) and 27 (bad health status).

Perception of parenting was measured based on three items from the Parent Questionnaire that are used in child health clinics [31]: enjoyment in parenting, self-confidence as a parent, and support in parenting by their (ex-)partner. Statements were answered on a 4-point scale (1 = totally incorrect; 4 = totally correct). Cronbach’s alpha appeared to be low (0.52); items were therefore reported separately. 

(3) Self-reported healthcare utilization (T1 and T2) was assessed with yes/no items from the NL-TiC-P questionnaire [32,33], including (1) the number of telephone calls or visits made to the general practitioner, child health clinic, and pediatrician, (2) number of hospital day treatments and admissions, and (3) emergency department visits, and the frequency of such visits [32]. For T1, children born preterm were excluded because they were expected to have more healthcare utilization on average. For the TP and FP cases, one visit to the pediatrician for the evaluation of their abnormal NBS result was excluded from the calculation.

Background characteristics of participants were collected, including sex, age, marital status, education level, country of birth (the Netherlands or another country), number of children and details about the child who underwent the NBS test (i.e., age, sex, whether the child was born preterm (<37 weeks) or at term (≥37 weeks), and birthweight (≤2500 or >2500 g). Participants were additionally asked to report the NBS result, and if applicable, the result of the second heel prick and follow-up testing at a hospital. 

### 2.5. Data Analysis

Descriptive analyses were used to provide an overview of sociodemographic and clinical characteristics. To compare cases and controls in terms of sociodemographic and clinical characteristics, (Likelihood ratio) chi-square tests and t-tests were utilized, as appropriate. To explore differences between participants with FP, TP, and IC results, Kruskall–Wallis and ANOVA analyses were conducted when applicable. For post-hoc analyses in cases of ANOVA, Tukey tests were employed. To address multiple testing, a conservative *p* threshold of <0.01 was considered statistically significant. All data analyses were performed using Statistical Package for the Social Sciences (SPSS), version 26.

## 3. Results

### 3.1. Study Population

**Figure 1:**
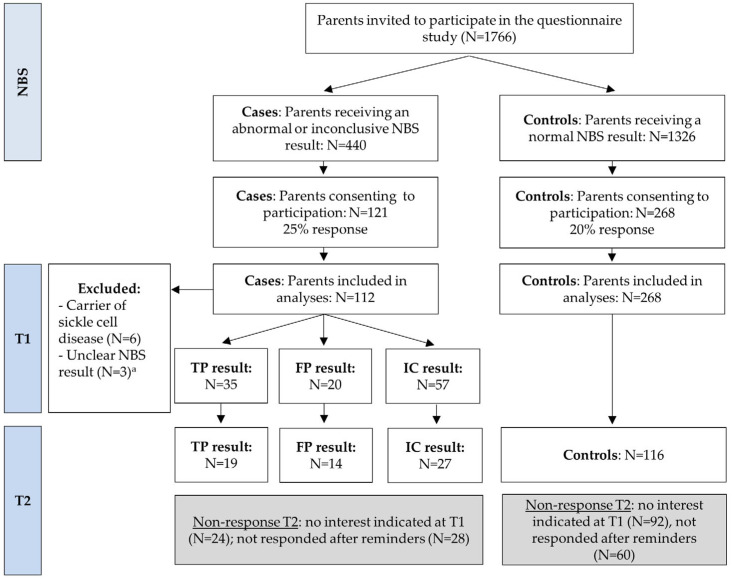
Flowchart of the study. Parents with an inconclusive result were invited for a second heel prick, which gave a normal result. T1 was sent 5 weeks after the NBS result and T2 was sent 4 months after the NBS result. NBS Newborn bloodspot screening; TP true-positive; FP false-positive; IC inconclusive. ^a^ In case the NBS result could not be derived from the questionnaire, the response was excluded from analyses.

In total, 1766 parents were invited to participate in the questionnaire study, of whom 389 agreed to participate (overall response rate = 22%, see Figure 1). Of those invited, 440 parents were asked to participate due to receiving an abnormal or inconclusive NBS result, with 121 consenting to participate (a response rate of 25% for this group, referred to as “cases”). Additionally, 1326 parents with a normal NBS result were invited, with 268 agreeing to participate (a response of 20% for “controls”). The data analyses for T1 included 112 cases (35 TP, 20 FP, and 57 IC) and 268 controls. Among the cases, 60 out of 112 (54%) completed the questionnaire T2 (19 TP, 14 FP, and 27 IC), while among the controls, 116 out of 268 (43%) completed T2. Parents with TP and FP results reported referrals for ten and nine different disorders, respectively, which largely reflected the case mix of the whole population. Parents with IC results gave different reasons for their results including a questionable result for one specific disease and insufficient blood collection or a blood transfusion.

Table 1 provides an overview of the characteristics of study participants. At T1, newborns in the cases group were, on average, significantly older (mean > 8 weeks) compared to those in the control group (7.6 weeks; *p* < 0.001). In addition, newborns in the cases group were less likely to be born at term (*p* = 0.002). Other characteristics were not significantly different between cases and controls. 

### 3.2. Perceptions of NBS

Table 2 outlines participants’ perceptions of various aspects of NBS for each subgroup at T1. In all groups, a majority of participants believed they had sufficient knowledge about NBS prior to participation. Notably, IC participants indicated significantly less often that they had sufficient knowledge compared to controls (*p* = 0.004; 74% vs. 78%, respectively). A lower proportion of TP and FP parents perceived having sufficient knowledge (57% and 65%, respectively); however, this was not statistically significant compared to controls. No significant differences were observed in participants’ attitudes towards NBS; 90% or more in all groups believed the heel prick test was beneficial for their child. However, FP participants were less likely to believe that the NBS was reliable compared to controls (*p* = 0.003; 60% vs. 93% respectively). In contrast, TP participants more often believed NBS was reliable compared to controls (*p* = 0.003; 100% vs. 93% respectively). At T1, one FP and one IC participant expressed regret about participating in NBS. None of the participants indicated that they would not intend to participate in the NBS program again. For all questions on parents’ perceptions, similar results were reported at T2 (data not shown).

Overall, 77% (27/35) of TP participants and 65% (13/20) of FP participants were satisfied with how they received the initial abnormal NBS result, and 89% (31/35) of TP and 85% (17/20) of FP participants were satisfied with the follow-up testing in a hospital. Among the 57 IC participants, 68% reported satisfaction with how they were informed about the extra heel prick test. Those who were not satisfied mainly mentioned a lack of information regarding the need for a second heel prick and the prompt visit from the screener without prior notice.

### 3.3. Psychosocial Impact of NBS Result

#### 3.3.1. Emotions Regarding Test Result at Different Time Points

**Figure 2:**
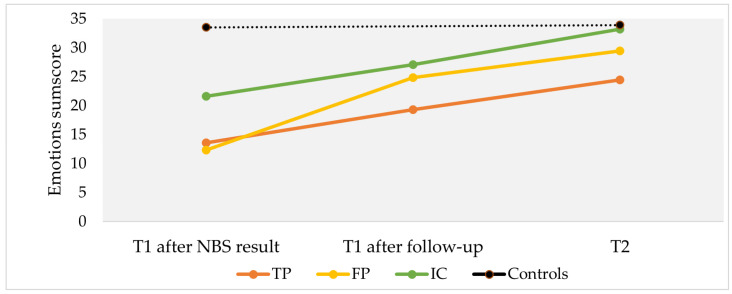
Mean sum scores of reported emotions regarding NBS test results at three different moments in time. Moments in time include (1) T1 after NBS result: after being informed about the (initial) NBS test result; (2) T1 after follow-up: after being informed about the (definitive) test result after follow-up, and (3) T2: at follow-up. Questionnaire T1 was sent 5 weeks after the NBS result and T2 was sent 4 months after the NBS result. Scores range from 0 (very negative emotions) to 35 (very positive emotions) about the NBS test result, with higher scores indicating more positive emotions (sum score of 7 items: feeling reassured, not startled, not worried, not anxious, happy, not angry, and relief). TP, true-positive; FP, false-positive; IC, inconclusive.

Emotions regarding the test result were assessed using mean sum scores of parents’ retrospectively reported emotions at three different moments in time (Figure 2). Participants with TP (mean sum score = 13.6, SD = 4.9), FP (mean = 12.3, SD = 3.9) and IC (mean = 21.8, SD = 9.4) reported more negative emotions after receiving the initial NBS result or the request of a second heel prick compared to controls (mean = 33.5, SD = 2.3) (all *p* < 0.001). After receiving definitive NBS results (reported at T1), all cases reported fewer negative emotions. At T2, TP (mean = 24.4, SD = 4.9) and FP (mean = 29.5, SD = 8.6) participants experienced more negative emotions compared to controls (mean sum score = 33.9, SD = 1.6; both *p* < 0.001). 

**Figure 3:**
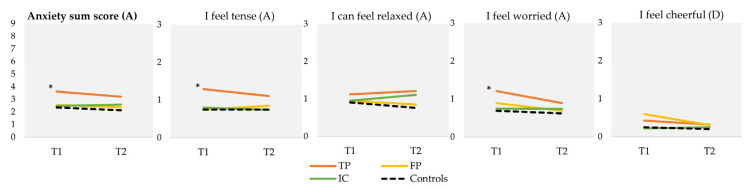
Mean scores on HADS anxiety (A) and depression (D) items at T1 and T2, and HADS anxiety sum score. Scores on individual items range from 0 to 3, with a lower score indicating less anxiety or depressive symptoms. “I can feel relaxed” and “I feel cheerful” concern reversed items. For the sum score, scores range from 0 to 9, with a lower score indicating less anxiety. T1 was sent 5 weeks after the NBS result and T2 was sent 4 months after the NBS result. * Statistically significant difference between TP and controls at T1 based on a *p*-value of <0.01 (ANOVA). HADS Hospital Anxiety and Depression Scale; NBS Newborn bloodspot screening; TP, true-positive; FP, false-positive; IC, inconclusive.

TP participants scored significantly higher on the HADS anxiety items “I feel tense” (mean = 1.29, SD = 0.91) and “I feel worried” (mean = 1.21, SD = 0.81) at T1 compared to controls (mean = 0.75, SD = 0.60, *p* < 0.001; mean = 0.69, SD = 0.65, *p* < 0.001, respectively) (Figure 3). Consequently, the HADS anxiety sum score was also significantly higher in TP participants (mean sum score = 3.6 (out of a maximum score of 9, based on three HADS anxiety items), SD = 2.2), compared to controls (mean sum score = 2.36, SD = 1.0; *p* < 0.001). No significant differences were found on the single depression item or between the groups at T2. 

#### 3.3.2. Perceptions of Child Vulnerability, Child’s Well-Being, and Parenting

No significant differences were observed in parental perceptions of child vulnerability between TP (median (min–max) = 7 (4–14)), FP (median (min–max) = 6 (4–10)), IC (median (min–max) = 6 (4–15)), and control participants (median (min–max) = 6 (4–15)) (tested with Kruskal–Wallis test due to non-normality) at either T1 or T2.

Concerning parent-reported child’s health status (tested with ANOVA), no significant differences were observed between TP (mean = 14.1, SD = 2.7), FP (mean = 13.8, SD = 1.9), and IC (mean = 14.0, SD = 2.7) participants compared to controls (mean = 13.8, SD = 2.3) at T1. At T2, TP (mean = 14.0, SD = 2.4), FP (mean = 13.3, SD = 2.7) and IC (mean = 14.4, SD = 2.6) participants again showed similar responses compared to controls (mean = 14.0, SD = 2.4). No significant differences were found. 

Table 3 shows participants’ responses to statements on perceptions of parenting. No differences were observed between the four groups at T1 or T2.

### 3.4. Healthcare Utilization

Regarding healthcare utilization, TP and FP participants with children born at term reported significantly more frequent visits to the pediatrician for their newborns compared to controls (*p* < 0.001 and *p* = 0.007, respectively) at both T1 and T2, as shown in Table 4. Similarly, TP and FP participants reported more frequent hospital day treatments for their newborns compared to controls at T1 (*p* < 0.001 and *p* = 0.003, respectively). At T2, only TP participants reported more frequent hospital day treatments compared to controls (*p* < 0.001). FP participants reported more frequent hospital admissions than controls at T1 (*p* = 0.005), but this difference was no longer observed at T2. At T2, TP participants reported more frequent hospital admissions compared to controls (*p* = 0.002). TP and IC participants visited the emergency department more often than controls at T1 (*p* < 0.001 and *p* = 0.006 respectively), but these differences were no longer significant at T2. 

## 4. Discussion

This study aimed to assess the psychosocial impact and reported healthcare utilization among parents with abnormal and inconclusive NBS results compared to those with negative (normal) NBS results. To our knowledge, a comparison of the psychosocial impact among parents who receive different types of NBS results covering a variety of disorders has not received much attention [11,19,34], with most studies focused on only one group of results (e.g., FP) or one specific disorder, for example, cystic fibrosis [18,20]. In addition, research on psychosocial impact of inconclusive results with repeated testing is lacking. The study revealed several important findings.

Overall, the study found positive attitudes towards NBS and relatively low psychosocial impact among all groups of parents, with most negative emotions being transient. Understandably, parents who received FP NBS results were less likely to perceive NBS as reliable. Additionally, parents with a TP, FP, or IC result reported significantly more negative emotions compared to controls in the short term, with this negativity persisting for TP and FP parents in the long term. Parents with a TP result also exhibited significantly higher anxiety post-NBS. These findings align with the notion that receiving a positive NBS result causes distress and negative emotions, especially for TP parents. 

These results are consistent with some previous research [5,11,35], but differ from others. For example, O’Connor et al. [19] found no differences in psychological impact among parents receiving a TP, an FP, or a negative result at 4–6 months post-NBS. Meanwhile, our results are in line with previous reports suggesting a significant psychological impact for parents with an FP result in the form of increased stress and worry [11,34], although it seems relatively short-lived [5,8]. Comparing psychosocial impacts across different types of NBS results and a variety of disorders is complex due to variations in outcomes and a lack of harmonization of measurement time points in previous studies.

Adequate explanation of the NBS result by healthcare providers is crucial to support parents’ understanding and limit both short- and long-term negative psychological impacts, especially for FP cases. A significant portion of FP parents in our study (1/3) reported dissatisfaction with how the initial abnormal result was provided. Improving communication methods tailored to parents’ cultural and educational backgrounds and preferences is suggested to enhance the NBS experience [5,36,37]. 

We did not identify differences in spousal support nor in other perceptions of parenthood between subgroups compared to controls. In contrast, a previous study [19] suggested an FP result might have a negative impact on spousal relationships, while a different qualitative study also suggested that the impact of an FP result may strengthen the relationship [12]. 

Despite initial negative emotions and some dissatisfaction with the process, especially regarding the need to repeat the NBS for cases involving inconclusive results, all parents across the TP, FP, and IC subgroups expressed their willingness to participate in NBS again in a subsequent pregnancy. This supports previous reports [5] and may reflect an understanding of the importance of NBS [35], especially for parents with a true-positive result, and their appreciation of the well-organized follow-up care. 

TP and FP parents reported a higher utilization of healthcare services, including visits to pediatricians and hospital care, compared to controls. We observed no differences with regard to visits to the general practitioner or child health clinic. This corresponds to previous studies, indicating increased hospital care utilization among FP cases [13,14,15,16]. A previous US cohort study showed that more preterm infants with false-positive NBS results experienced a higher frequency of acute outpatient visits, in contrast to term infants [21]. In our study, we excluded preterm children with regard to short-term healthcare use. 

In the control group, we observed a relatively high proportion of parents reporting that they had seen a pediatrician (34% at T1), who may have been confusing a specialized pediatrician with the youth healthcare physician that most children see at the child health clinic.

IC parents reported more visits to the emergency department in the short-term compared to controls, possibly due to increased worry among parents resulting from initial unclear results or conditions or complications underlying an inconclusive NBS result [38]. Despite the higher use of healthcare among FP and IC cases, our study did not find any differences in parental perceptions of child vulnerability and of the child’s health status.

This study has strengths and limitations. A strength is the comparison of the psychosocial impact of different NBS results (TP, FP, and IC) on parents, providing crucial information that can help improve the NBS experience. The results of this study have been presented to the Dutch government and policy makers. Despite differences between countries, there are many similarities in the way NBS is organized and the study’s findings may inform other NBS programs worldwide. Limitations include potential inaccuracies in self-reporting of NBS results and healthcare utilization. The aim of this study was to explore the psychosocial impact of a broad scope of possible effects. Therefore, not all questions or scales were validated (in Dutch) and some were specifically constructed for the study, which limits accuracy and comparison to other studies. The diversity within the IC subgroup, the time gap between cases and controls in completing the T1 questionnaire, and the relatively low follow-up response rate were also observed as limitations. The majority of respondents were highly educated and of Dutch origin. Additionally, the recruitment method restricted the ability to send reminders, which may have limited participation and possibly introduced response bias, also skewing the participant population towards higher levels of education. More research is needed among parents with lower levels of educational attainment and of non-Dutch origin. Qualitative studies may be helpful to better interpret the outcomes and psychosocial impact of various NBS results on parents.

## 5. Conclusions

This study revealed a predominantly temporary higher psychosocial impact on parents with true-positive, false-positive, and inconclusive NBS results compared to controls. Healthcare utilization, especially pediatrician and hospital visits, was significantly higher among parents with true-positive and false-positive parents in the short-term, with TP parents, not unexpectedly, continuing to utilize healthcare at a higher rate in the long-term, but also FP parents reporting more pediatric visits in the long-term. We found no significant differences in parent-reported child vulnerability, perceptions of child health status, or views of parenthood. Despite the impact on psychosocial functioning and healthcare utilization, all parents expressed their willingness to participate in the NBS again. This study’s findings can inform NBS programs on how to optimize support and address the needs of families receiving different NBS results.

## Figures and Tables

**Table 1:** Characteristics of participants.

	Total *N* = 380	Cases *N* = 112			Controls *N* = 268	*p*-Value ^c^
**Characteristics of the parent(s) ^a^**		TP *N* = 35	FP *N* = 20	IC *N* = 57		
T1 questionnaire filled in by, *n* (%)						0.829
Mother	301 (79)	32 (91)	15 (75)	40 (70)	214 (80)	
Father	31 (8)	0 (0)	2 (10)	8 (14)	21 (8)	
Mother and father	46 (12)	3 (9)	3 (15)	9 (16)	31 (11)	
Missing	2 (1)	0 (0)	0 (0)	0 (0)	2 (1)	
Age parent completing questionnaire, mean (SD)	32.0 (4.2)	32.0 (4.8)	32.5 (4.3)	32.2 (4.7)	32.0 (4.1)	0.661
Marital status, *n* (%)						0.529
Single	10 (3)	0 (0)	1 (5)	1 (2)	8 (3)	
Married/living together	367 (96)	35 (100)	18 (90)	54 (95)	260 (97)	
Missing	3 (1)	0 (0)	1 (5)	2 (3)	0 (0)	
Education level, *n* (%)						0.298
Low	12 (3)	2 (5)	0 (0)	3 (5)	7 (3)	
Middle	115 (30)	16 (46)	5 (25)	17 (30)	77 (29)	
High	250 (66)	17 (49)	14 (70)	36 (63)	183 (68)	
Missing	3 (1)	0 (0)	1 (5)	1 (2)	1 (0)	
Both parents born in the Netherlands, *n* (%)	329 (87)	28 (80)	15 (75)	49 (86)	237 (88)	0.101
Number of children ^b^, mean (SD)	1.8 (0.9)	1.9 (1.3)	1.8 (1.0)	1.6 (0.7)	1.8 (0.9)	0.515
**Characteristics of the newborn**						
Average age in weeks at T1, mean (SD)	7.8 (1.9)	8.5 (2.2)	8.3 (1.9)	8.4 (2.2)	7.6 (1.7)	<0.001 ***
Average age in weeks at T2, mean (SD)	22.8 (4.4)	22.1 (3.0)	23.7 (3.9)	25.5 (7.2)	22.2 (3.5)	0.030
Female, *n* (%)	176 (46)	19 (54)	7 (35)	22 (39)	128 (48)	0.153
Born at term: ≥37 weeks, *n* (%)	347 (91)	33 (94)	16 (80)	45 (79)	253 (94)	0.002 **
Birth weight: >2500 g, *n* (%)	354 (93)	33 (94)	17 (85)	51 (89)	253 (94)	0.128

^a^ At baseline (T1; first questionnaire). ^b^ Number of children in total, including the newborn that was tested. ^c^ *p*-value of significance tests between cases and controls with chi-square tests for categorical variables and t-tests for continuous variables. Statistically significant difference between cases and controls based on a ** *p*-value of <0.01; *** *p*-value of <0.001. TP, true-positive; FP, false-positive; IC, inconclusive.

**Table 2:** Participants’ perceptions of NBS at T1.

Question		Response 4–5 ^a^ *N* (%)	Median (Min–Max)	*p*-Value (Compared to Controls) ^d^
Sufficient knowledge prior to the heel prick (1 = certainly not, 5 = certainly) ^b^	TP	20 (57)	4 (1–5)	0.025
	FP	13 (65)	4 (1–5)	0.038
	IC	42 (74)	4 (1–5)	0.004 **
	Controls	210 (78)	4 (1–5)	
(Positive) Attitude towards the heel prick (1 = bad, 5 = good)	TP	34 (97)	5 (3–5)	0.724
	FP	18 (90)	5 (1–5)	0.754
	IC	56 (98)	5 (3–5)	0.702
	Controls	263 (98)	5 (3–5)	
(High) Perceived reliability of the heel prick (1 = not reliable, 5 = reliable)	TP	35 (100)	5 (4–5)	0.003 **
	FP	12 (60)	5 (1–5)	0.003 **
	IC	48 (84)	5 (2–5)	0.275
	Controls	249 (93)	5 (2–5)	
Regret about the heel prick (1 = no regret, 5 = a lot of regret) ^c^	TP	0 (0)	1 (1–3)	0.663
	FP	1 (5)	1 (1–5)	<0.001 ***
	IC	1 (2)	1 (1–4)	0.036
	Controls	0 (0)	1 (1–2)	
Intention (not) to participate in the heel prick again (1 = certainly, 5 = certainly not) ^c^	TP	0 (0)	1 (1–1)	0.410
	FP	0 (0)	1 (1–3)	0.060
	IC	0 (0)	1 (1–3)	0.293
	Controls	0 (0)	1 (1–2)	

^a^ All participants who answered 4 or 5 on a scale of 1–5. ^b^ Self-perceived knowledge. ^c^ Reverse scoring. ^d^ *p*-value of significance tests between cases and controls based on independent-samples Kruskal–Wallis testing. Statistically significant difference between cases and controls based on a ** *p*-value of <0.01 or *** *p*-value of <0.001. NBS Newborn bloodspot screening; TP, true-positive; FP, false-positive; IC, inconclusive.

**Table 3:** Agreement with statements on parenting at T1 and T2.

Statements		T1			T2		
		Response 3–4 ^a^ *N* (%)	Median (Min–Max)	*p*-Value ^b^ (Compared to Controls)	Response 3–4 ^a^ *N* (%)	Median (Min–Max)	*p*-Value ^b^ (Compared to Controls)
I enjoy parenting	TP	34 (97)	4 (2–4)	0.448	19 (100)	4 (3–4)	0.341
	FP	20 (100)	4 (3–4)	0.497	13 (100)	4 (3–4)	0.715
	IC	55 (98)	4 (1–4)	0.531	27 (100)	4 (3–4)	0.333
	Controls	266 (99)	4 (2–4)		114 (98)	4 (2–4)	
I feel confident in being a parent	TP	32 (91)	3 (2–4)	0.115	18 (95)	3 (2–4)	0.338
	FP	19 (95)	3 (2–4)	0.790	13 (100)	3 (3–4)	0.694
	IC	54 (96)	3 (2–4)	0.527	26 (96)	3 (1–4)	0.776
	Controls	251 (94)	3 (1–4)		111 (96)	3 (2–4)	
I feel supported in parenting by my partner	TP	35 (100)	4 (3–4)	0.505	19 (100)	4 (3–4)	0.139
	FP	20 (100)	4 (3–4)	0.182	13 (100)	4 (3–4)	0.894
	IC	56 (100)	4 (3–4)	0.322	27 (100)	4 (3–4)	0.250
	Controls	262 (98)	4 (1–4)		112 (97)	4 (1–4)	

^a^ All participants who answered 3 or 4 on a scale of 1–4; (mostly/totally) correct. ^b^ *p*-value of significance tests between cases and controls based on independent-samples Kruskal–Wallis testing. TP, true-positive; FP, false-positive; IC, inconclusive.

**Table 4:** Self-reported healthcare utilization per subgroup.

Health Care Provider		T1 Only Children Born at Term ≥37 Weeks			T2 All Children		
		≥1 Visit ^a^ *N* (%)	Median (Min–Max)	*p-*Value ^b^	≥1 Visit ^a^ *N* (%)	Median (Min–Max)	*p-*Value ^b^
General practitioner	TP	21 (64)	1 (0–12)	0.088	13 (72)	2 (0–50)	0.192
	FP	8 (50)	0 (0–6)	0.447	9 (69)	2 (0–15)	0.128
	IC	19 (42)	0 (0–20)	0.903	18 (67)	1 (0–15)	0.523
	Controls	121 (48)	0 (0–6)		71 (61)	1 (0–10)	
Child health clinic	TP	32 (97)	2 (0–5)	0.218	17 (94)	3 (0–7)	0.164
	FP	15 (94)	2 (0–3)	0.972	12 (92)	5 (0–6)	0.197
	IC	42 (93)	2 (0–5)	0.885	27 (100)	4 (1–6)	0.050
	Controls	249 (98)	2 (0–6)		116 (100)	3 (2–8)	
Pediatrician	TP	23 (70)	2 (0–19)	<0.001 ***	14 (74)	4 (0–19)	<0.001 ***
	FP	10 (63)	1 (0–8)	0.007 **	9 (69)	2 (0–9)	<0.001 ***
	IC	21 (47)	0 (0–20)	0.087	14 (52)	1 (0–24)	0.111
	Controls	85 (34)	0 (0–4)		43 (37)	0 (0–10)	
Day treatment hospital	TP	16 (49)	0 (0–6)	<0.001 ***	9 (50)	0 (0–10)	<0.001 ***
	FP	7 (44)	0 (0–4)	0.003 **	6 (46)	0 (0–7)	0.050
	IC	14 (31)	0 (0–20)	0.022	4 (15)	0 (0–7)	0.989
	Controls	32 (13)	0 (0–6)		18 (16)	0 (0–6)	
Hospital admission	TP	10 (30)	0 (0–3)	0.092	10 (53)	0 (0–7)	0.002 **
	FP	7 (44)	0 (0–8)	0.005 **	5 (39)	0 (0–1)	0.168
	IC	16 (36)	0 (0–8)	0.127	8 (30)	0 (0–3)	0.163
	Controls	42 (17)	0 (0–2)		22 (19)	0 (0–7)	
Emergency department	TP	10 (31)	0 (0–1)	<0.001 ***	7 (37)	0 (0–2)	0.032
	FP	4 (25)	0 (0–2)	0.016	3 (23)	0 (0–1)	0.545
	IC	10 (22)	0 (0–2)	0.006 **	6 (22)	0 (0–3)	0.322
	Controls	15 (6)	0 (0–2)		17 (15)	0 (0–3)	

^a^ All participants who reported at least one appointment/admission with the given healthcare provider. For the TP and FP cases at T1 one visit to the pediatrician (referral for evaluation of their abnormal NBS result) was excluded. At T1, children born preterm (<37 weeks) were excluded. ^b^ *p*-value of significance tests between cases and controls based on independent-samples Kruskal–Wallis testing. Statistically significant difference between cases and controls based on a ** *p*-value of <0.01; *** *p*-value of <0.001. TP, true-positive; FP, false-positive; IC, inconclusive.

## Data Availability

The datasets are not publicly available due to privacy reasons but are available from the corresponding author upon reasonable request.

## Supplementary Materials

The following supporting information can be downloaded at: https://www.mdpi.com/article/10.3390/ijns10010018/s1, S1 Questionnaire.
