# Supplementary material for: Psychosocial Impact of a True-Positive, False-Positive, or Inconclusive Newborn Bloodspot Screening Result: A Questionnaire Study among Parents
Source: Int J Neonatal Screen. 2024 Mar 5;10(1):18. doi: 10.3390/ijns10010018 (PMC10971090; doi:10.3390/ijns10010018)
Supplement: Supplementary file 1 [file IJNS-10-00018-s001.zip › IJNS-2861260-supplementary.pdf]

Supplementary Material

Article: van den Heuvel et al. Psychosocial impact of a true positive, false positive or inconclusive newborn bloodspot screening test result: a questionnaire study among parents.

Questionnaire used in the study

This is a questionnaire about the heel prick test. When a baby is a few days old, he or she will be offered a heel prick test. With the heel prick test a few drops of blood are taken from the baby's heel.

With the heel prick test, we find out if a baby has a rare but serious health condition. Early treatment can improve the baby's health, and prevent severe disability or even death. The Dutch government pays for and arranges the heel prick.

We would like to hear your opinion about the heel prick test and the result of the heel prick test.

Thank you for participating!

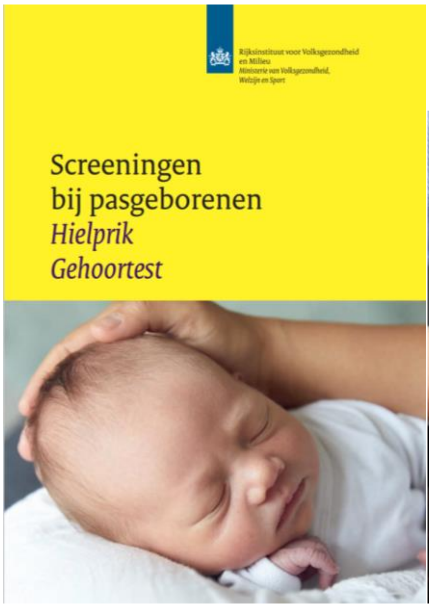

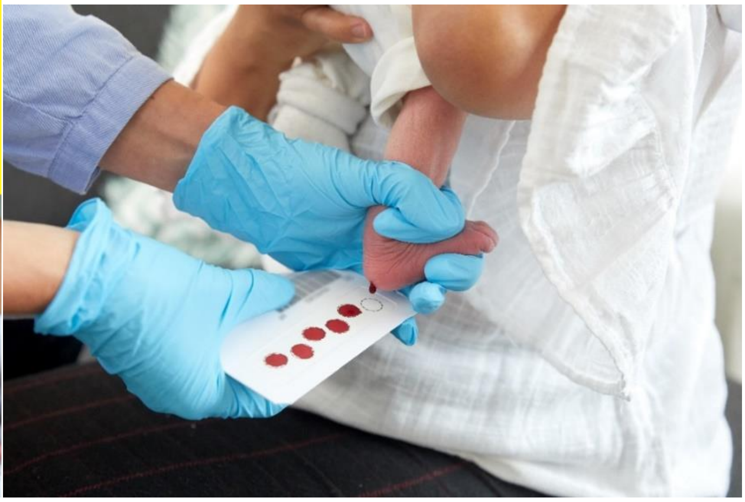

Who is completing this questionnaire? Please tick the correct box:

- ☐ Mother
- ☐ Father
- ☐ Mother and Father
- ☐ Someone else, namely (specify):………………………………………

A. Your opinion about the heel prick test

With the following questions we would like to know your opinion about the heel prick test. Tick the box:

a. Did you have enough knowledge about the heel prick before the heel prick test was carried out?

Certainly not	☐	☐	☐	☐	☐	Certainly

b. I find the heel prick test for my baby:

Bad	☐	☐	☐	☐	☐	Good

c. How reliable do you think the heel prick test is?

Not reliable	☐	☐	☐	☐	☐	Reliable

d. Do you regret participating in the heel prick test?

A lot of regret	☐	☐	☐	☐	☐	No regret

e. If you are ever pregnant again, would you participate in the heel prick test?

Certainly not	☐	☐	☐	☐	☐	Certainly

B. Results of the heel prick test

The following questions are about the results of the heel prick test of your child.

1. Can you describe the test result of the heel prick test of your child in your own words? What does this test result mean and what consequences does this test result have for you and your child?
………………………………………………………………………………………………
………………………………………………………………………………………………

2. How did you feel when you heard the first heel prick test result?

	1	2	3	4	5	
Reassured	☐	☐	☐	☐	☐	Not reassured
Frightened	☐	☐	☐	☐	☐	Not frightened
Worried	☐	☐	☐	☐	☐	Not worried
Scared	☐	☐	☐	☐	☐	Not scared
Unhappy	☐	☐	☐	☐	☐	Not unhappy
Angry	☐	☐	☐	☐	☐	Not angry
Relieved	☐	☐	☐	☐	☐	Not relieved

3. What was the result of the first heel prick test of your child?
- ☐ I was notified that the results were **normal** *[continue with question 19]*
- ☐ I was notified that my child is a **carrier of sickle cell diseas**e *[continue with question 19]*
- ☐ My child first needed an **extra heel prick test** *[continue with question 4]*
- ☐ The (irst result was immediately **abnormal** and we had to go to the doctor [*continue with question 8]*
- ☐ I do not want to answer this question *[continue with question 19]*

4. Why was an extra heel prick test necessary?
- ☐ The blood sample was **too small** for the laboratory
- ☐ My child had a **blood transfusion** before the heel prick test was carried out
- ☐ I was notified that the result of the heel prick test was **not yet clear**
- ☐ I don't know

5. Are you satisfied about the way you were informed about the extra heel prick test?
- ☐ Yes
- ☐ No, because ………………………………………………………………………………..

6. Has your child had an extra heel prick test in the mean time?
- ☐ Yes
- ☐ No, the extra heel prick test has not been carried out yet *[continue with question 19]*
- ☐ No, because I did not want an extra heel prick test *[continue with question 19]*

7. What was the result of the extra heel prick test?
- ☐ The results were **normal** *[continue with question 18]*
- ☐ The results were**abnormal** *[continue with question 8]*
- ☐ I am still **waiting** for the results. *[continue with question 19]*
- ☐ My child is a **carrier of sickle cell disease** *[continue with question 18]*
- ☐ I do not want to answer this question *[continue with question 19]*

8. Who informed you about the **abnormal** heel prick test result and that follow-up investigations are required?
- ☐ The general practitioner (family doctor)
- ☐ The paediatrician (who works in the hospital)
- ☐ Someone else, (please specify) …………………………………………………

9. How satisfied are you about the way the health professional informed you about the abnormal heel prick test results? *(If you want, you can specify your answer)*
- ☐ Satisfied, because……………………………………………………………… ………………………………………………………………………………….
- ☐ A little satisfied, because………………………………………………………. ………………………………………………………………………………….
- ☐ Not satisfied, not dissatisfied, because………………………………………. ………………………………………………………………………………….
- ☐ A little dissatisfied, because…………………………………………………… ………………………………………………………………………………….
- ☐ Dissatisfied, because…………………………………………………………… ………………………………………………………………………………….

10. Do you think that the health professional was knowledgeable about the condition?
- ☐ Yes
- ☐ A little
- ☐ No, because (please specify)…………………………………………………………

…………………………………………………………………………………………

11. Did the health professional have enough time for you to answer your questions?
- ☐ Yes
- ☐ A little
- ☐ No

After you received the abnormal results, you went **to the hospital** with your child for **further investigations with the paediatrician**. The following questions are about this follow-up visit.

12. Did your child have any further investigations? (Explanation of further investigations: children with abnormal heel prick test results are further examined in the hospital. This is carried out in the care of a paediatrician)
- ☐ Yes
- ☐ No, not yet *[continue with question 19]*
- ☐ I don't know *[continue with question 19]*
- ☐ Other, (specify)……………………… *[continue with question 19]*

13. How satisfied are you about the follow-up investigations in the hospital?
- ☐ Satisfied
- ☐ A little satisfied
- ☐ Not satisfied, not dissatisfied
- ☐ A little dissatisfied
- ☐ Dissatisfied

14. Was there enough time during follow-up investigations in the hospital for your question to be answered?
- ☐ Yes
- ☐ A little
- ☐ No

15. What were the results of the further investigations?
- ☐ Your child has a condition that was detected through the heel prick test
- ☐ Your child does not have the condition after all
- ☐ The results are not yet known *[continue with question 19]*
- ☐ I prefer not to answer this question *[continue with question 19]*
- ☐ Other, (specify) …………………………………… *[continue with question 19]*

16. How much time was there between the moment that you were **notified** about the abnormal heel prick result and the **results** of the follow-up investigations in the hospital? (0 days means that you received the result of the follow-up investigations on the same day that you heard that the heel prick test was abnormal)
- ☐ ……. weeks and/or ……. days
- ☐ I don't know

17. What is your opinion about the time frame between the abnormal results and the results of the follow-up investigations?

- ☐ Good
- ☐ Too long
- ☐ Too short

18. How did you feel when you heard the final results?

	1	2	3	4	5	
Reassured	☐	☐	☐	☐	☐	Not reassured
Frightened	☐	☐	☐	☐	☐	Not frightened
Worried	☐	☐	☐	☐	☐	Not worried
Scared	☐	☐	☐	☐	☐	Not scared
Unhappy	☐	☐	☐	☐	☐	Not unhappy
Angry	☐	☐	☐	☐	☐	Not angry
Relieved	☐	☐	☐	☐	☐	Not relieved

19. If you have any remarks about your experience with the heel prick test result, you can write them down here:

……………………………………………………………………………………………………

……………………………………………………………………………………………………

……………………………………………………………………………………………………

C. How are you and your child's health?

The following questions are about the health of your child, the care your child received after birth and your well-being as a parent. We are curious how parents experienced this after the heel prick.

1. Below you find statements about your child. For each statement please indicate to what extent the statement applies to your child. Please tick one box only for each statement.

	Completely incorrect	Mostly incorrect	Mostly correct	Completely correct
a. In general my child seems less healthy than other children.	☐	☐	☐	☐
b. I often think about calling the doctor about my child	☐	☐	☐	☐

c. When there is something going around, my child usually catches it	☐	☐	☐	☐
d. I often check on my child at night to make sure that he / she is okay. .	☐	☐	☐	☐

The questions below are about the **general health of your child from birth up until now**. You can answer the questions by ticking the answer that best suits your child.

2. Since birth, has your child had:

	Never	Sometimes	Often
Colic (abdominal cramps)?	☐	☐	☐
Shortness of breath?	☐	☐	☐
Itching or a skin rash?	☐	☐	☐
Fever?	☐	☐	☐

	Never	Sometimes	Often
3. Did your child sleep restlessly	☐	☐	☐
4. Did your child cry during the night?	☐	☐	☐
5. Did your child have a poor appetite?	☐	☐	☐

6. How often has your child been happy or tense since birth?

	Never	Sometimes	Often
Happy	☐	☐	☐
Tense	☐	☐	☐

Below you will find a number of questions that relate to how you feel. For each question, tick the answer that best describes *how you have felt during the* ***past week***. Try not to think too long about your answer.

7. I feel tense:

☐ Usually
☐ Often
☐ Sometimes
☐ Not at all

8. I can sit quietly and relax

☐ Definitely
☐ Usually
☐ Not often
☐ Not at all

9. I am often anxious / worried
- ☐ Very often
- ☐ Often
- ☐ Sometimes
- ☐ Rarely

10. I feel cheerful
- ☐ Not at all
- ☐ Not often
- ☐ Sometimes
- ☐ Usually

11. How do you feel about parenthood?

	Completely incorrect	Mostly incorrect	Mostly correct	Completely correct
a. I enjoy parenthood	☐	☐	☐	☐
b. I feel confident as a parent	☐	☐	☐	☐
c. I feel supported by my (ex-)partner in the upbringing of our child	☐	☐	☐	☐

D. Health care use from birth to now

We would like to know with which doctors you have had an appointment for your newborn child.

Which appointments count?

- Regular checkups for your child
- Appointments because your child had physical complaints
- Appointments where the doctor made a home visit to your child
- Telephone advice from the doctor (or doctor's assistant) for your child
- Phone calls with the ‘prescription telephone line’ for your child

Which appointments do <u>not</u> count?

- Phone calls to make an appointment
- Appointment for others such as yourself or another child

1. How many appointments did your child have with the general practitioner (family doctor) since birth?

- ☐ None at all
- ☐ .... appointments (specify)

Count all appointments your child had since birth. If you can’t remember exactly how many appointments your child has had, then write down how many there were more or less. **Please note:** look carefully at the list above to see which appointments count**.**

2. How many appointments did your child have with preventive child health services (JGZ) / well baby clinics/child health clinic (consultatiebureau)?

- ☐ None at all
- ☐ ...... appointments (specify)

3. Did your child have an appointment with the paediatrician since birth?

- ☐ No
- ☐ Yes, number of appointments (specify):………

4. Has your child been to the hospital for treatment during the day? *Your child was treated as an out-patient and did not stay the night.*

- ☐ No
- ☐ Yes, …………times (specify)

5. Has your child ever been hospitalized since birth? *Your child had to stay the night in hospital. For example because it needed an operation and could not go home immediately.*

- ☐ No [***continue with question 7]***
- ☐ Yes, …………times (specify)

6. How long did your child spend in hospital? *Has your child been in hospital more than once, then add all the days together.*

…… days in total (specify)

7. Since birth, how often did your child need the Accident & Emergency department (EHBO) in the hospital?

- ☐ Never
- ☐ ...... times (specify)

8. Has your child been on any medication since birth?

- ☐ No
- ☐ Yes, namely (specify which medication)…………………………………………………

9. Is your child on a diet? (For example does your child drink special milk?)

- ☐ No
- ☐ Yes, namely (specify)…………………………………………………………………

E. A few final questions about you

The following questions are about yourself. We ask these questions to see whether the parents who complete the questionnaire are comparable to parents in general.
In case you fill in this questionnaire together with your partner, we ask you, if possible, to enter *the mother's* details below.

1. My age is ….. years

2. I am:
   - ☐ Married
   - ☐ Cohabitant
   - ☐ Single
   - ☐ Other,(specify) :…………………………………………………………..

3. My education:
   - ☐ Primary Education
   - ☐ Technical and Vocational Training
   - ☐ Lower and Middle General Secondary Education
   - ☐ Middle Vocational Education
   - ☐ Higher General Secondary Education
   - ☐ Pre-university Education
   - ☐ Higher Vocational Education
   - ☐ University
   - ☐ I don't know
   - ☐ Other, (specify):

4. The mother of the new born baby was born in:
   - ☐ The Netherlands
   - ☐ Turkey
   - ☐ Morocco
   - ☐ Surinam
   - ☐ Dutch Antilles/Aruba
   - ☐ Other, (specify) ……………………………………………

5. The father of the new born baby was born in:
   - ☐ The Netherlands
   - ☐ Turkey
   - ☐ Morocco
   - ☐ Surinam
   - ☐ Dutch Antilles/Aruba
   - ☐ Other, (specify)……………………………………………..

6. Do you have any other children?
   - ☐ No
   - ☐ Yes, number of children (specify):……..

The following questions are about your new born baby:

7. How old is your child at the moment (or your children, if they are twins)?
   You can enter the age in weeks or in months.
   …….. weeks (specify)
   ………months (specify

8. What is the sex of your baby?
- ☐ Boy
- ☐ Girl
- ☐ Twins (twins, triplets etc): …… boys ….... girls (specify numbers)
- ☐ Other (specify)…………………………………………..

9. After how many weeks pregnancy was your baby (or were your twins) born?
- ☐ Less than 37 weeks
- ☐ 37 weeks or more
- ☐ I don't know

10. What was the birth weight of your baby (or twins)?
- ☐ 2500 gram or less
- ☐ More than 2500 gram
- ☐ I don't know

11. Do you have any final remarks about this questionnaire or about the heel prick? Please write them down below:

Participation gift voucher

Among all parents who have completed the questionnaire, 10 gift vouchers of € 10 will be raffled. Write your e-mail address below if you want to join the raffle for a gift voucher.

………………………………………………………………………………

Your e-mail address will only be used to send you the voucher and will be deleted directly afterwards. Your e-mail address will be saved separately from the answers.

Participation short questionnaire

With your permission, we would like to send you a shorter questionnaire about the heel prick in three months' time. With your answers we can improve the heel prick. Filling in this short questionnaire takes 5-10 minutes. We will then draw another 10 gift vouchers of €10,-.
Do you want to answer a few more questions about the heel prick in three months' time?

o Yes, the questionnaire can be sent to the following email address or house address

______

o No

Your (e-mail) address is only used to send this questionnaire to you. You may then still choose whether or not to participate. Your e-mail address will be saved separately from your answers.

Thank you for completing the heel prick test questionnaire!

Your answers are stored in a safe place and will be processed anonymously.

If you have any further remarks or questions about this questionnaire then please contact us via the following mail address: hielprikvragenlijst@tno.nl

[Don't forget to click 'Send answers' before closing the window.]

If you have any questions or comments about this questionnaire, please contact us via e-mail address: hielprikvragenlijst@tno.nl.
